# YA is needed for proper nuclear organization to transition between meiosis and mitosis in *Drosophila*

**DOI:** 10.1186/1471-213X-9-43

**Published:** 2009-07-23

**Authors:** Katharine L Sackton, Jacqueline M Lopez, Cindy L Berman, Mariana F Wolfner

**Affiliations:** 1 Dept of Molecular Biology and Genetics, Cornell University, Ithaca NY 14853, USA

## Abstract

**Background:**

The *Drosophila *YA protein is required to initiate the embryonic cleavage divisions. After egg activation, YA enters nuclei and interacts with chromatin and the nuclear lamina. This study was designed to define more precisely the events prior to the first cleavage division that are dependent upon YA.

**Results:**

We find that meiosis is completed normally in the absence of YA function. The first defects in embryos and eggs from mutant mothers first appear just after the completion of meiosis, and are seen as abnormal associations among the resultant haploid nuclei. These defects are associated with asynchronies in the cell cycle-dependent chromatin condensation state of the haploid nuclei. However, we find evidence of DNA replication in the absence of YA function.

**Conclusion:**

Our data suggest YA function is needed at a control point, following meiosis II and the initiation of the first postmeiotic S phase, which is sensitive to the chromatin condensation state of the haploid meiotic products.

## Background

Mature *Drosophila *oocytes are arrested in metaphase of meiosis I. To begin development, oocytes must undergo a number of changes that are collectively called egg activation [1-5]. The egg is hydrated, proteins in its vitelline membrane undergo cross-linking, certain maternal RNAs are polyadenylated and translated while others are degraded [6], the phosphorylation state of many proteins changes [7-9], the cortical actin cytoskeleton is reorganized [10], and meiosis resumes. Egg activation in *Drosophila *[1] and other insects [11-13], is independent of fertilization (in contrast to the situation in other animals) [14]; it is triggered instead by passage through the female's reproductive tract. Despite differences in trigger, the initial cause of egg activation in essentially all animals appears to be an increase in intracellular calcium [4,5,15]. Upon activation, *Drosophila *oocytes complete meiosis rapidly without cytokinesis, resulting in four haploid nuclei located near the membrane and aligned perpendicular to the long axis of the egg [16,17]. The chromosomes of all four meiotic products decondense and appear morphologically to be in a state similar to interphase [18]. In unfertilized, activated eggs, all four meiotic products synchronously replicate their DNA once, and then condense their chromosomes ([19], B. Loppin personal communication). The four nuclei then associate into a single composite polar body, which appears as a rosette-shaped array of condensed chromosomes [20,3].

In fertilized eggs, the innermost meiotic product (the nucleus furthest from the egg cortex) usually becomes the maternal pronucleus and remains decondensed, while the remaining three meiotic products associate into a polar body near the surface of the egg [18,21]. The sperm nucleus undergoes reorganization to become the paternal pronucleus. Its chromatin decondenses and recruits maternally provided proteins, such as YA [22,23]. The maternal and paternal pronuclei migrate towards each other and closely appose (align next to each other) for the first mitotic division. This migration requires microtubules nucleated at the male pronucleus and microtubule associated proteins such as Ncd (non-claret disjunctional), KLP3A (kinesin-like protein at 3A), and Asp (abnormal spindle protein) [16,24,25]. The first S phase of all the five haploid nuclei in embryos likely occurs just prior to or concurrent with apposition, as assessed by staining with the S-phase marker PCNA (Proliferating Cell Nuclear Antigen) [19]. The two pronuclear genomes subsequently divide on a shared spindle, but because remnants of the nuclear envelope remain around the pronuclei, the parental genomes remain separate through anaphase; this unusual first mitosis is called the "gonomeric" division.

Chromosomes derived from the male and female pronuclei finally mix together during telophase of the gonomeric division, resulting in two diploid zygotic nuclei [17,21]. These zygotic nuclei then undergo thirteen rapid mitotic divisions without cytokinesis. During these cycles, S and M phases are normally tightly coupled. However, mutation of any of three maternal effect genes *plutonium (plu), giant nuclei (gnu)*, and *pan gu (png) *results in repeated rounds of replication (S phase) without mitosis, resulting in giant nuclei [26,27]. These three genes and *fs(1)Ya *appear to function specifically at this unique cell cycle transition from meiosis to mitosis, having no known functions in any adult tissues or other stages of development.

The embryos produced by females homozygous for null or strong hypomorphic mutations of the gene *fs(1)Ya *fail to initiate the first embryonic mitosis [28,29]. YA, the product of this gene, is a novel protein with a developmentally regulated subcellular localization [28]. In oocytes, YA is cytoplasmic, but in early embryos or in activated eggs that are not fertilized, YA localizes to the nuclear lamina and nucleoplasm, where it associates with lamin, DNA, and histone H2B [9,30]. The nuclear lamina is the proteinaceous inner layer of the nuclear envelope that provides structure to the nucleus, organizes the chromatin and provides nucleation sites for chromosome condensation [31-33]. In this paper, we analyze in detail the developmental arrest that occurs in embryos deficient for YA (hereafter called *Ya*^2 ^embryos), in an effort to understand which events in early development depend upon YA. We show that YA function is not required for egg activation, but rather immediately thereafter for the transition from meiosis to mitosis. We find that meiotic segregation occurs normally in *Ya*^2 ^eggs and embryos, and that the resultant nuclei can afterwards enter S phase. However, without YA function, female meiotic products undergo inappropriate associations with one another and with the male pronucleus. These nuclei appear to have variable chromatin condensation states, particularly as seen in asynchronies in the phosphorylation state of histone H3 and localization of PCNA. Our data suggest that YA protein function is required for early embryos to transit a control point between post-meiotic DNA replication and the initiation of the first mitotic division.

## Methods

### Drosophila strains

The null allele *fs(1)Ya*^2 ^[28] was carried in stock as *X^X*, *y^2^ fs(1)Ya^2^ w^bf^ spl sn^3^/Y, y^+^ fs(1)Ya^+^w^+^B *(abbreviated as *X^X*, *Ya*^2 ^and *Y, Ya^+^*, respectively). For FISH studies and immunofluorescence, YA-deficient eggs or embryos (referred to here as "*Ya*^2 ^eggs" or "*Ya*^2 ^embryos", respectively) were generated from *X^X*, *Ya*^2 ^females carrying a normal Y chromosome (daughters of females from the stock crossed to Oregon R P2 males; Oregon R P2 is a wild-type strain of flies with a reduced tendency to hold mature eggs [34]). For FISH, control eggs and embryos were obtained from *X^X*, *Ya*^2^/*Y, Ya^+ ^*females from the stock. For immunofluorescence, Oregon R P2 embryos were used as the control. For collections of unfertilized eggs for FISH, we used the same mutant combinations, but crossed into the stocks a transgene to induce expression of the *Drosophila *sex peptide (*hs-SP *[35]) to stimulate production and deposition of large numbers of eggs. The histone H3-FLAG transgenic stock was a gift from B. Loppin and is described in Loppin et al. [19]. YA-deficient embryos expressing the H3-FLAG transgene in a heterozygous state were generated by crossing H3-FLAG males to females of the *Ya*^2 ^stock described above. For collections of unfertilized eggs for immunofluorescence, *X^X*, *Ya*^2 ^females carrying a normal Y chromosome were mated to sons-of-*tudor *(see below). The *tudor *stock is *tudor*^1^*bw sp/CyO *[36]. For in vitro egg activation experiments, unfertilized eggs were obtained from *X^X*, *Ya*^2 ^females; controls were eggs from Oregon R P2 [34]. Flies were raised at room temperature (≈ 23°C) with a 12:12 hour light: dark cycle on yeast-glucose media.

### FISH analysis

#### Egg/embryo collection

Three day old *X^X*, *Ya*^2^/*Y, Ya^+^*; *hs-SP *or *X^X*, *Ya*^2^/*Y*; *hs-SP *virgin females were heat shocked for 30 minutes at 37°C to induce expression of *hs-SP *[35], and returned to 25°C to recover for 2.5 hours before 0–15 minute collections of unfertilized control and *Ya*^2 ^mutant eggs, respectively, were undertaken. *X^X*, *Ya2/Y, Ya^+^*females were mated to *shi*^1^/*Y, Ya^+^*males for 0–15 minute collections of control embryos. Mating *shi*^1^/*Y, Ya+ *males with *X^X*, *Ya*^2^/*Y *females yielded *Ya*^2 ^mutant embryos.

#### Hybridization and microscopy

Probes specific for three different chromosomes were prepared and used as in [37,38]. To detect the Y chromosome, a Y-chromosome specific (AATAC)n [39] repeat was labeled with Rhodamine-4-dUTP. To detect the X chromosome, a 1.688 satellite sequence [40] specific to the X chromosome was labeled with fluorescein-dUTP. The maternal *X^X *and the paternal X are each seen as one signal with the 1.688 satellite probe. The 1.688 satellite probe also hybridizes to the Y, *Ya^+^*chromosome, because the region of the X that is translocated to the Y chromosome contains the 1.688 satellite. Thus, we could distinguish between the maternal and paternal pronuclei in the mutant embryos fertilized by Y, *Ya^+^*-bearing sperm: such embryos have both a maternal Y (because the mothers are X^X/Y) recognized only by the fluorescein Y probe, and a paternal Y, *Ya^+^*chromosome, which hybridizes to both the X and Y chromosome probes. To detect chromosome 2, the histone gene repeat [41] was labeled with biotin-16-dUTP, and hybridization signals were detected by incubating the eggs and embryos with Cy5-conjugated streptavidin.

Embryos and eggs were fixed using the procedure described in Lopezet al. [23] and stored in methanol at 4°C. The fixed samples were DAPI-stained following hybridization as described in [37]. We observed distinct and separate DAPI-stained regions representing the individual nuclei; we report the number of nuclei per egg or embryo based on these data. FISH and DAPI staining of eggs and embryos were analyzed on a Zeiss Axiovert 10 microscope attached to a Bio-Rad MRC600 confocal imaging system; DAPI by epifluorescence and FISH signals by confocal on the same egg or embryo, taken sequentially in the same sitting.

We refer to nuclei that are a single DAPI-stained entity containing associated sets of chromosomes by the genomic copy number, i.e. "triploid" or "diploid", based on the FISH signal for the chromosome 2 probe, although we have not confirmed fusion of the nuclear envelopes that would distinguish a true nuclear fusion product from a cluster of very closely associated nuclei.

### Immunofluorescence

X^X, *Ya*^2^/Y or Oregon R P2 females were mated to Oregon R P2 males for 0–15 minute collections of *Ya*^2 ^mutant or control embryos. Laid unfertilized eggs were collected from unmated females or females mated to spermless males (sons of Oregon R P2 males × *tud*^1^*bw sp *females) [36]. Embryos fixed in methanol/heptane were rehydrated in phosphate-buffered saline with 0.1% Triton X-100 (PBST) [42], and anti-phospho histone H3 (Upstate) or anti-PCNA (gift of PA Fisher, SUNY Stonybrook) [43] were used at 1:100 or 1:50 dilutions, respectively, in PBST. Anti-rabbit secondary was Alexa 488-conjugated (Invitrogen, Carlsbad, CA). DNA was stained with 10 μg/ml propidium iodide or a 1:750 dilution of Oligreen (Invitrogen) stock solution. Eggs or embryos laid by H3-FLAG control or X^X, *Ya*^2^/Y; H3-FLAG mutant females were fixed as above, and anti-FLAG (Sigma) was used at a 1:400 dilution, with Alexa 633-conjugated anti-mouse secondary antibody (Invitrogen). For immunofluorescence detection of anti-PH3 or anti-PCNA, fixed and stained samples were mounted in 75% glycerol containing 940 mM *n*-propyl gallate. For immunofluorescence with anti-FLAG, samples were washed in MeOH and mounted in 2:1 benzyl benzoate: benzyl alcohol [44]. Eggs and embryos were analyzed using confocal microscopy (Leica TCS SP2 system equipped with an argon-krypton laser and coupled to a Leica DMRBE microscope). Leica software was used to collect images. Where appropriate, Leica software was used to project multiple optical sections into a single image and to overlay images. Because estimating nuclear ploidy by size and DNA intensity is confounded by the asynchronous condensation states of the nuclei, without FISH we cannot correlate asynchronous cell cycle state and abnormal nuclear associations.

### Preparation and analysis of in vitro activated oocytes

Oocytes isolated from 400 to 1200 virgin females were obtained by blender agitation followed by serial sieve purification, according to [3] with minor modifications. Oocytes thus isolated were activated by incubation in hypotonic buffer (Activation Buffer) [2,3]. As previously described, total activation time is measured from the onset of incubation in Activation Buffer to the transfer of eggs to fixative. During egg activation, the vitelline membrane becomes cross-linked and the egg becomes opaque by visual inspection [3]. Opacity and bleach-resistance [1,2] of the vitelline envelope, and the presence of post-metaphase I stages indicated successful activation of eggs. Eggs were DAPI-stained, visualized by microscopy, and assigned to meiotic stages by comparison to data presented in [3,17].

## Results and discussion

### YA-deficient unfertilized eggs complete meiosis upon egg activation

**Figure 1:**
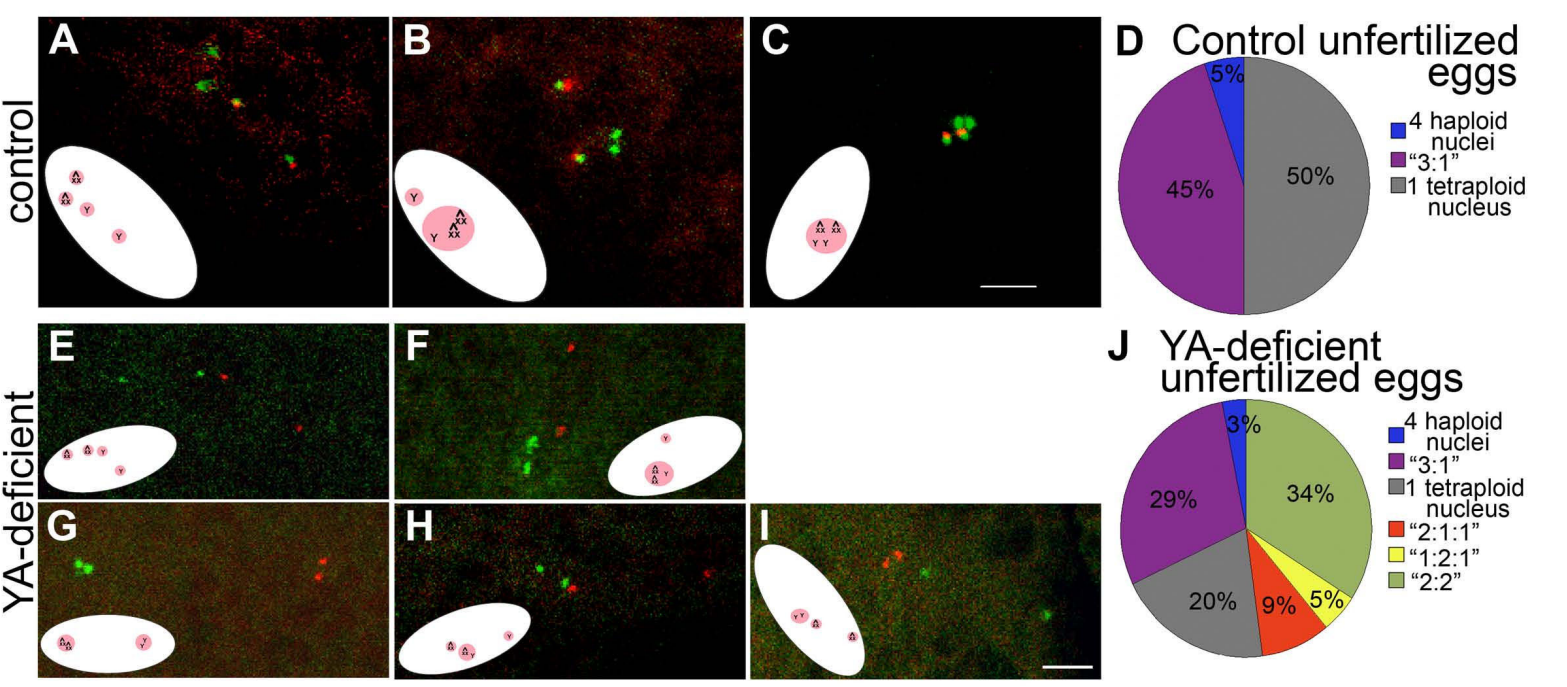
**Meiotic product behavior in control and YA-deficient unfertilized eggs**. *In situ *hybridization to the X and Y chromosomes in 0–15 minute old unfertilized eggs laid by X^X/Y, *Ya*^+ ^(control) females (A-C) and X^X *Ya*^2^/Y (*Ya*^2 ^mutant) females (E-I). The X chromosome probe's signal is green, and the Y chromosome's is red. The green hybridization signal to the X chromosome also recognizes the maternal Y, *Ya*^+ ^in control eggs (thus the Y, *Ya*^+ ^is red and green in A-C). The X probe does not recognize the maternal Y chromosome in YA-deficient eggs (thus the Y is only red in E-I). (A-C) Unfertilized eggs containing (A) four separate haploid meiotic products, (B) one triploid nucleus with two X^X chromosomes and one Y chromosome, the result of association of three meiotic products, and one haploid Y-containing nucleus. (C) all four meiotic products associated. (D) Chart of percentages of nuclear distributions observed in the 22 control eggs. (E-I) YA-deficient unfertilized eggs containing (E) four haploid meiotic products, (F) one triploid nucleus with two X^X chromosomes and one Y chromosome, reflecting three associated meiotic products, and a separate haploid Y chromosome-containing nucleus, (G) two diploid nuclei, one containing two X^X chromosomes and the other containing two Y chromosomes. H and I both contain three DAPI-stained nuclei: (H) one Y-containing haploid nucleus, one diploid nucleus containing one X^X and one Y chromosome and one haploid X^X-containing nucleus, (I) two haploid X^X chromosome-containing nuclei and one diploid nucleus containing two Y chromosomes. (J) Chart of percentages of nuclear distributions observed in the 38 *Ya*^2 ^eggs. DAPI staining clearly showed the number of distinct nuclei per egg (four in A, E, two in B, F, G, one in C, three in H, I). Inset illustrations in each panel show the orientation of the egg and positions of DAPI stained nuclei inside (not drawn to scale). Bar = 4 μm for all panels.

As a control for our analysis of YA-deficient eggs, we used FISH to examine the chromosome complements of the meiotic products in control unfertilized eggs. We observed three different patterns (Figure 1; second chromosome data not shown). (1) In 5% of eggs, four individual haploid meiotic products are seen (Figure 1A). (2) In 45% of eggs three nuclei are associated, with one haploid nucleus remaining separate (type "3:1") (Figure 1B). (3) In 50% of eggs, all four haploid nuclei have associated (Figure 1C) to form a rosette-shaped array of condensed chromosomes. As described above, this third pattern represents the normal terminal arrest of activated, unfertilized eggs [3,20,29]. Together the three nuclear distribution patterns in the control laid unfertilized eggs reflect normal chromosome segregation during meiosis (forming four haploid nuclei), and subsequent associations of first three and then all four of those haploid nuclei with one another to ultimately form the large tetraploid inactive nucleus called the polar body.

If YA function is not required for completion of meiosis, then we expect to see these normal nuclear distributions in laid unfertilized YA-deficient eggs, but if YA function is required for separation of chromosomes in meiosis, we might expect only a tetraploid nucleus or two diploid nuclei. In eggs laid by mothers homozygous for the null *Ya*^2 ^allele, DAPI fluorescence analysis reveals four or fewer abnormally condensed nuclei [29]. The position of the nuclei in the anterior of the *Ya*^2^ eggs and their orientation relative to the eggs' long axis appear normal. As in control eggs, a small percentage of *Ya*^2 ^eggs show four DAPI-stained nuclei, each of which is haploid (Figure 1E). Also as in controls, some YA-deficient eggs have a single condensed nucleus showing hybridization signals for the complements of all four meiotic products (unpublished observation), although the frequency of these eggs is lower than in controls. Some *Ya*^2 ^eggs also contain two DAPI-stained nuclei of the "3:1" type; as in control eggs, the larger nucleus has a triploid DNA content and the smaller has a haploid content (Figure 1F). Overall, the presence of a haploid nucleus in the "3:1" pattern, and the presence of eggs that show four haploid nuclei, together demonstrate that meiotic chromosome segregation occurs properly and is completed in the absence of YA function. The frequencies with which the three different normal nuclear distributions are observed vary somewhat between control and YA-deficient laid unfertilized eggs (Figure 1D&1J), possibly because nuclear associations may occur more slowly in the absence of YA function.

### Many meiotic products in YA-deficient unfertilized eggs exhibit abnormal associations

In addition to the normal classes of nuclear distribution just described, two abnormal nuclear distributions are seen in eggs from *Ya*^2^females. First, ~34% of the *Ya*^2 ^eggs contain two diploid nuclei ("2:2" type) (Figure 1G); this phenomenon is never seen in wildtype eggs. One of these diploid nuclei contains two X^X chromosomes, while the other has two Y chromosomes. We never saw "2:2" type eggs in which any one nucleus contained both an X^X and a Y chromosome. Therefore, these nuclei likely derive from a failure to undertake or complete meiosis II or from abnormal association of sister nuclei following meiosis completion. We believe the latter explanation is correct because we can distinguish two individual copies of each chromosome in each associated pair of meiotic products as two FISH signals; if these eggs were arrested before anaphase II, the paired sister chromatids of a given homolog would reveal only one signal [37]. Since the DAPI staining does not reveal the eggs to be in anaphase, we believe that these signals are of sister chromatids that segregated in meiosis II and are then present together after association of the meiotic products.

Second, ~14% of the *Ya*^2 ^eggs have three DAPI stained nuclei. These eggs all contain one nucleus with a diploid content of chromosomes, and two more that are haploid (Figure 1H, I), but this nuclear distribution can further be divided into two subtypes. Eggs of the first subtype contain a diploid nucleus containing two second chromosomes, one X^X and one Y chromosome; and two haploid nuclei each with one second chromosome and one of the remaining sex chromosomes (Figure 1H). In this "1:2:1" subclass, the diploid nucleus is always positioned between the two haploid nuclei. The distribution of nuclei in these eggs demonstrates that meiosis II has been completed but that the central two nuclei, each products of a separate meiosis II spindle, have subsequently associated. In the second subtype of eggs with three nuclei, one nucleus is diploid and contains either two X^X chromosomes or two Y chromosomes, and two nuclei are haploid, each containing one of the remaining sex chromosomes (Figure 1I). In these "2:1:1" eggs the {X^X X^X} or {Y Y} diploid nuclei are never found between the two haploid nuclei; they are always one of the outer nuclei of the three (that is, either the nucleus closest to the egg cortex or the nucleus closest to the center of the egg). The "2:1:1" nuclear distribution could result from failure of segregation in one of the two meiotic spindles of meiosis II. However, given the "1:2:1" nuclei also observed in *Ya*^2 ^eggs, the simplest explanation is that meiosis completes normally, but subsequently two resulting haploid meiotic products then associate or fuse (See Additional file 1 – Figure S1).

Every category of associated nuclei observed in *Ya*^2 ^eggs requires, or is at least consistent with, normal completion of meiosis. Our results thus all suggest that completion of meiosis does not require YA function, but that lack of YA function does disrupt proper associations between the nuclei produced by meiosis.

### Meiosis completes upon in vitro activation of YA-deficient unfertilized eggs

**Figure 2:**
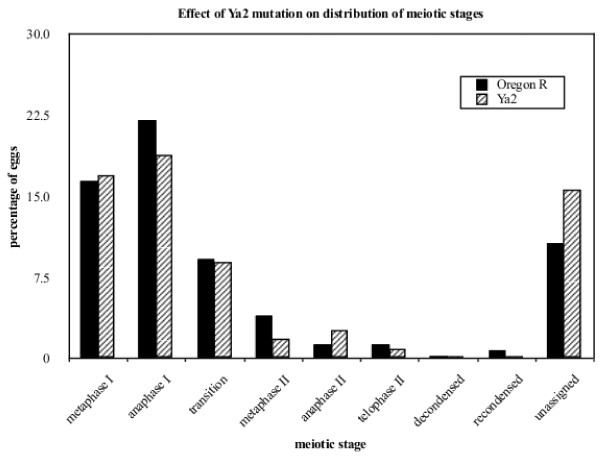
**Effect of Ya^2 ^mutation on distribution of meiotic stages**. Wildtype (Oregon R P2) and X^X, *Ya*^2^/Y (*Ya*^2 ^mutant) eggs were activated and staged based on DAPI staining. Eggs with apparently condensed disorganized chromatin are grouped into the "unassigned" category, although some of the eggs in this category likely had completed meiosis. There are no statistically significant differences in the distribution of stages of wildtype vs. YA-deficient eggs as assessed by Fisher's exact test; for each of the nine categories of meiotic stages, comparison of the fraction of wildtype eggs in a stage to the fraction of YA-deficient eggs in that stage gave p values ranging from p = 1 to p = 0.061. Wildtype total n = 327 eggs. *Ya*^2 ^total n = 280 eggs.

We used in vitro activation to test independently whether meiosis can complete in *Ya*^2 ^eggs. As observed by Page & Orr-Weaver [3], in vitro activation of a pool of oocytes results in a range of meiotic stages depending on the length of incubation in hypotonic buffer (See Additional file 1 – Figure S2). For oocytes incubated for 15 +/- 1 minutes we found similar distributions of meiotic stages from *Ya*^2^ females and wildtype females, indicating that progression through meiosis is not dependent on YA function (Figure 2). Furthermore, we found that the proportion of postmeiotic oocytes increased with length of incubation at the same rate for *Ya*^2 ^and wildtype oocytes (data not shown). The meiotic figures in YA-deficient eggs visualized by DNA and microtubule fluorescence furthermore appear comparable to wildtype (data not shown). Thus, we conclude that YA is not necessary for proper completion of meiosis.

Multiple events of egg activation (other than the completion of meiosis) also occur in fertilized laid *Ya*^2 ^embryos (see Additional file 1 – Figure S3), including crosslinking of the vitelline membrane [1], SMAUG translation [45], and GNU and ERK dephosphorylation [7,8]. We conclude that in the absence of YA function, *Drosophila *eggs can activate, including resuming and completing meiosis. However, based on the phenotypes seen in laid, unfertilized *Ya*^2 ^eggs, their haploid nuclei do not behave normally once both meiotic divisions have been finished.

### Nuclei in YA-deficient embryos exhibit abnormal associations

Proper nuclear associations are not critical in activated unfertilized eggs, since these eggs are laid but do not develop in *D. melanogaster*, a non-parthenogenic species of *Drosophila*. However, improper nuclear associations are almost certain to disrupt the development of fertilized eggs. We thus used FISH to examine the nuclear association phenotype in control and *Ya*^2 ^embryos.

**Figure 3:**
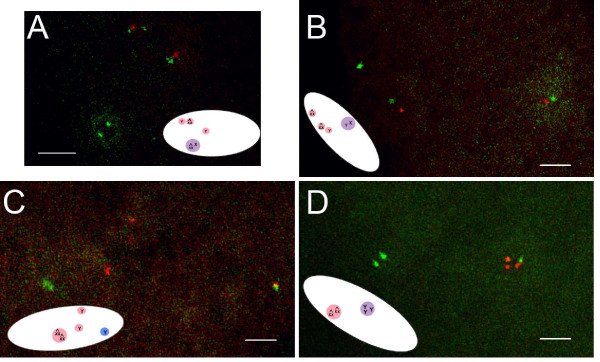
**Meiotic product behavior in control and YA-deficient embryos**. In situ hybridization to X and Y chromosomes in 0–15 minute old embryos from X^X/*Ya^+^*Y (control) and X^X *Ya^2^*/Y (*Ya^2^*mutant) mothers mated to X/Y, *Ya+*males. Inset illustrations in each panel show the orientation of the embryo and positions of DAPI stained nuclei inside (not drawn to scale). Pink circles represent maternally derived nuclei, blue circles represent paternally derived nuclei. The X chromosome probe's signal is green, and the Y chromosome probe's is red. The paternal Y chromosome was Y, *Ya^+^*, and therefore is marked with both red and green signals. (A) In the control gonomeric-stage embryo shown, the polar body nuclei (one haploid maternal X^X chromosome-containing nucleus and two haploid maternal Y chromosome-containing nuclei) have not yet begun to associate. The gonomeric nucleus consists of a maternal X^X and paternal X chromosome, each seen as a single dot with the X probe. Control n = 28 embryos. (B-D) YA-deficient embryos with (B) two haploid X^X chromosome-containing nuclei and one haploid Y chromosome-containing nucleus (all of maternal origin), and one diploid nucleus which is a product of an association between a male *Ya^+^*Y chromosome-containing pronucleus and a haploid X^X chromosome-containing nucleus of maternal origin, (C) one diploid X^X-containing nucleus (of maternal origin), two haploid Y-containing nuclei (of maternal origin) and a *Ya^+^*Y containing male pronucleus, (D) one diploid X^X chromosome-containing nucleus (of maternal origin) and one triploid nucleus which is presumably a product of an association between a diploid Y chromosome-containing nucleus (or two Y-containing haploid nuclei) of maternal origin and a *Ya^+^*Y chromosome-containing male pronucleus. *Ya2 *n = 54 embryos. Bars = 4 μm for all panels.

Immediately after fertilization in wildtype embryos, the sperm nucleus decondenses, assembles a YA-containing nuclear envelope and becomes closely apposed to the maternal pronucleus with which it enters the gonomeric division [18,22,46,47]. It has previously been described [47,48] that prior to this first mitotic division, the maternal nuclei are arranged in patterns similar to those of unfertilized control eggs shown in Figure 1 and described above. However, we did not observe any "pre-gonomeric" embryos among our 0–15 minute control embryo collections, probably because meiosis is completed very rapidly after fertilization (which occurs internally in *Drosophila*). Instead, the wildtype embryos we saw in our collections had three polar bodies and a diploid nucleus, presumably the gonomeric region, which contains the female and male pronuclei; one such embryo, a female, is shown in Figure 3A.

As with *Ya*^2 ^eggs, some (18%) of *Ya*^2 ^embryos resemble controls (Figure 3B), having a "gonomeric" nucleus with a haploid maternal content and a haploid paternal content, and three haploid maternally derived nuclei. *Ya*^2 ^embryos sometimes also have five separate haploid nuclei (four maternally-derived, one paternally-derived), like those of very early wildtype embryos (data not shown). However, in a majority of *Ya*^2 ^embryos, non-wildtype nuclear arrangements occur. In 52% of embryos, the male pronucleus does not associate with a maternal nucleus (Figure 3C). Embryos with five separate haploid nuclei were fixed very shortly after meiosis completion and fertilization, so the male pronucleus would not normally yet have associated with a maternal nucleus in these embryos, but in fixed embryos where any other nuclear associations have occurred, there should have been time before fixation for the male pronucleus to associate with the female pronucleus. Some of these embryos contain a tetraploid nucleus derived entirely from maternal products, while others contain both haploid and diploid nuclei (data not shown and Figure 3C, respectively). In 13% of *Ya*^2^ embryos, the male pronucleus has associated with a maternally-derived diploid nucleus, the latter likely reflecting one of the 2:2 abnormal associations seen in unfertilized eggs. For example, Figure 3D shows a triploid nucleus composed of two maternally derived pronuclei (each with a Y-chromosome) and a paternally derived pronucleus (with the paternal Y-chromosome). This sort of association is never seen in wildtype. Finally, 17% of the *Ya*^2 ^embryos are in various stages of deterioration [29], with fragmented or dramatically decondensed nuclei such that the number of chromosomes to which the probes bound was difficult to determine (see Additional file 1 – Figure S4).

The chromosome complements of *Ya*^2 ^embryo nuclei are consistent with the idea that after fertilization, meiosis is completed, the same abnormal nuclear associations as we previously found in unfertilized *Ya*^2 ^eggs can occur, and then the male pronucleus may or may not join with one of the resultant maternal nuclei. In over half the embryos the male pronucleus does not associate with a maternally-derived nucleus, while in other cases the male pronucleus associates inappropriately with groups of maternally-derived haploid nuclei. These results suggest that in the absence of YA function, the maternally- and paternally-derived haploid nuclei either fail to develop their identity (as a polar body versus a pronucleus), or that nuclei without YA are defective in the processes that normally ensure proper associations between the two pronuclei and among the three polar body nuclei. YA's localization to the nuclear lamina is potentially consistent with a role either in establishing pronuclear identity or in governing pronuclear apposition. Since there normally are some differences between pronuclei, such as distinct chromatin methylations or acetylations observed in mice [49], the mouse AKAP95 protein being specific to the female pronucleus [50], or the *Drosophila *histone H3.3 and HIRA proteins that are only present in the male pronucleus [19], a protein such as YA that assists in pronuclear coordination prior to the first mitosis may be essential to proceeding on to embryogenesis. Our observation in this study that no *Ya*^2 ^embryos displayed signs of nuclear division past the gonomeric division, consistent with Lin and Wolfner [28] and Liu et al. [22], supports the model that YA is required for embryonic mitosis.

The abnormal nuclear associations in *Ya*^2 ^eggs and embryos are quite different from the lack of male pronuclear association due to defects in pronuclear migration that occurs in mutants of four genes that encode microtubule-related proteins (*KLP3A, asp, ncd*, and *polo *[24,25,16,48]). These mutants and *Ya*^2 ^share the phenotype of improper male pronuclear association. However, the *Ya*^2^phenotype does not include repeated haploid mitosis of the female polar bodies as seen in *ncd *or *polo *[16,48], nor is chromatin condensation in *Ya*^2 ^embryos normal as it is in *KLP3 *or *asp *embryos [24,25]. If these phenotypes were more similar, a pronuclear migration defect might be proposed to explain the failure of the male pronucleus to associate with a female pronucleus in roughly half of *Ya*^2 ^embryos. Given the additional differences between the phenotypes of mutant microtubule-related proteins and the *Ya*^2^phenotype, it seems likely that instead of YA functioning in regulating physical migration of the pronuclei, YA acting in the nuclear lamina to establish pronuclear identity may facilitate subsequent pronuclear apposition.

In addition to the nuclear association defects reported here, *Ya*^2 ^egg and embryo nuclei also have a phenotype of overcondensed chromatin [29]. This overcondensation observed by DAPI staining is especially apparent in nuclei of "3:1" mutant unfertilized eggs. The volume of their larger nucleus would be expected to be three times greater than that of a haploid nucleus, but instead these triploid nuclei often appear very small due to highly condensed chromatin (data not shown). Similar observations of overcondensed chromatin can be made for diploid and tetraploid nuclei in *Ya*^2 ^eggs and embryos. YA's localization to the nuclear lamina and chromatin [23,30] is potentially consistent with a role in governing chromatin condensation state.

### Meiotic products in some YA-deficient eggs and embryos are asynchronous in PCNA and PH3 distribution

Because *Ya*^2 ^embryos complete meiosis but do not undergo the first mitotic division, we wanted to determine more precisely the cell cycle characteristics of the nuclei of YA-deficient eggs and embryos. Nuclei in *Ya*^2 ^eggs and embryos collected and fixed shortly (within 15 minutes) after they are laid have various chromatin condensation states as visualized by DAPI or propidium iodide staining: condensed nuclei, decondensed nuclei, or both condensed and decondensed nuclei in the same egg/embryo. The *Ya*^2 ^eggs and embryos collected and fixed at later time points (1–2 hours after they are laid) have primarily highly condensed nuclei, and some degrading nuclei [29]. Although condensed chromatin is usually mitotic, the highly condensed chromatin in *Ya*^2 ^eggs and embryos does not always resemble that of mitotic nuclei. Thus, we used antibodies to phospho-histone H3 (PH3) to determine whether the mutant's nuclei contained a histone modification characteristic of mitotic chromatin [51], and we used antibodies against PCNA, a component of the replication fork, to identify replicating (S phase) chromatin [52].

**Figure 4:**
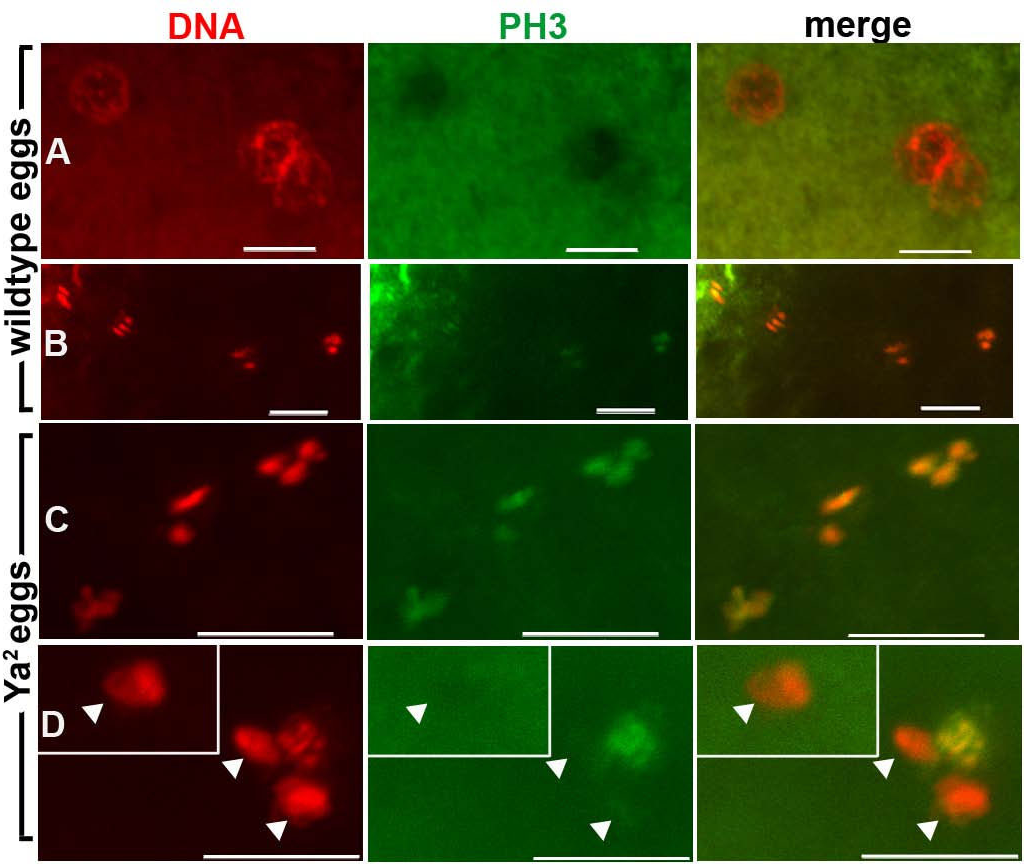
**Phospho-histone H3 distribution on chromatin of unfertilized wildtype and YA-deficient eggs**. Immunostaining of 0–15 minute old laid unfertilized wildtype and *Ya*^2 ^eggs (from Oregon R P2 and X^X *Ya*^2^/Y mothers, respectively). DNA shown in red, phospho-histone H3 (PH3) in green. A-C are projections of multiple confocal images. (A) Four decondensed wildtype nuclei in promeiotic interphase or S phase, with PH3 excluded from nuclei (any faint PH3 staining was around the nuclear periphery). (B) Four condensed wildtype meiotic products perpendicular to the egg cortex, with PH3 staining on all chromatin. (C) Condensed *Ya*^2 ^meiotic products (probably 1:1:2, post meiotic but just after telophase since still arranged linearly), with PH3 staining on all chromatin. (D) Four *Ya*^2 ^meiotic products in two planes; three grouped at the egg cortex and one deeper in (inset). Although all four nuclei have similar nuclear areas, and thus are likely all haploid, only one of the three haploid nuclei at the cortex forming the polar body has individualized chromosomes with PH3 staining. Arrowheads indicate decondensed nuclei without PH3 staining. Wildtype n = 64 eggs. *Ya*^2 ^n = 20 eggs. Bar = 10 μm.

**Figure 5:**
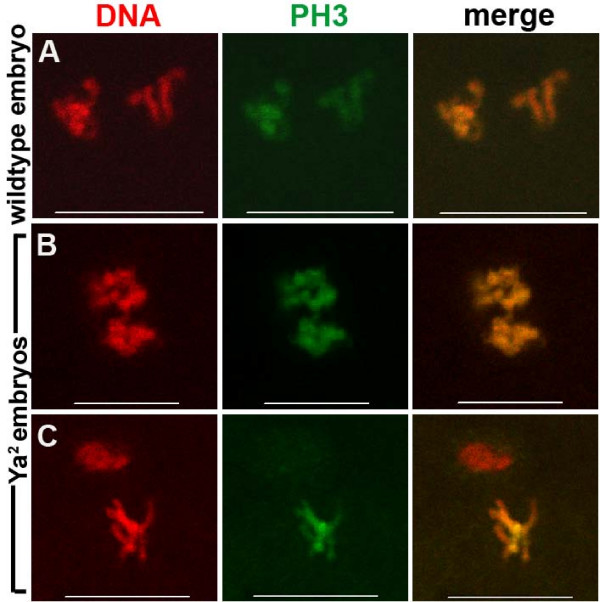
**Phospho-histone H3 distribution on chromatin of wildtype and YA-deficient embryos**. Immunostaining of 0–15 minute old wildtype and *Ya*^2 ^embryos. DNA shown in red, phospho-histone H3 in green. Embryos shown had three peripheral nuclei (not shown) and two nuclei apposed in the mid-anterior region. Apposed nuclei are shown. (A) Wildtype, (B) Synchronous apposed nuclei in a *Ya*^2 ^embryo (70% of *Ya*^2 ^embryos with apposed nuclei showed this phenotype), (C) Asynchronous apposed nuclei in a *Ya*^2 ^embryo (30% of *Ya*^2 ^embryos with apposed nuclei showed this phenotype). Wildtype n = 22 embryos with apposed pronuclei. *Ya*^2 ^n = 27 embryos with apposed nuclei. Bar = 10 μm.

**Figure 6:**
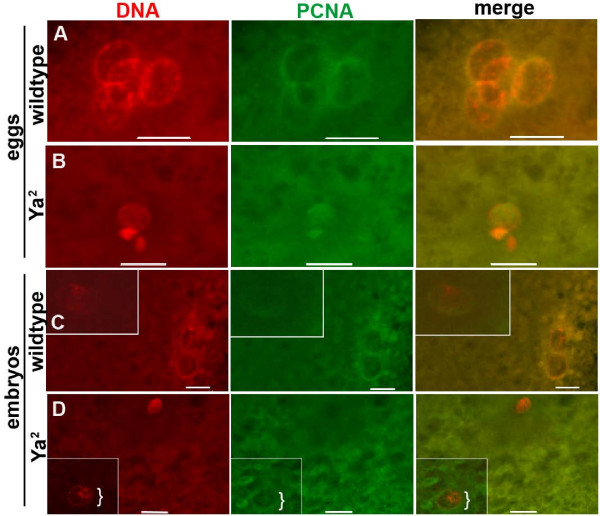
**PCNA distribution on chromatin of wildtype and YA-deficient eggs and embryos**. Immunostaining of 0–15 minute old laid unfertilized wildtype and *Ya*^2 ^eggs, and wildtype and *Ya*^2 ^embryos. DNA shown in red, PCNA in green. A and B are projections of multiple confocal images. (A) Four decondensed wildtype egg nuclei with PCNA on DNA, likely in S phase. 55% of wildtype eggs had PCNA staining nuclei, all synchronous. (B) Variously condensed *Ya*^2 ^egg meiotic products with PCNA staining on 2 out of 3 areas of chromatin. 26% of *Ya*^2 ^eggs had PCNA-positive nuclei; 2% were synchronous with PCNA, 24% were asynchronous. (C) Embryo with five decondensed wildtype meiotic products with PCNA staining on all. Two apposed pronuclei (inset) and three meiotic products forming a polar body (only two are visible in this plane). (D) *Ya*^2 ^embryo with two nuclei in two planes; one near the embryo cortex (inset) and one deeper in. Only one of the two has PCNA staining (bracket). Wildtype n = 42 eggs, n = 215 embryos. *Ya*^2 ^n = 34 eggs, n = 168 embryos. Bar = 10 μm.

The chromatin of condensed and overcondensed nuclei in *Ya*^2 ^eggs (Figure 4C) and embryos (Figure 5B) stain with PH3, as do condensed wildtype nuclei (Figure 4B, 5A), indicating that condensed or overcondensed *Ya*^2 ^chromatin is in a mitotic-like state. However, cell-cycle asynchrony of nuclear chromatin in a given egg or embryo is observed: in 25% of *Ya*^2 ^unfertilized eggs (Figure 4D) and 6% of *Ya*^2 ^embryos (Figure 5C), some nuclei are condensed and have PH3 staining, while one or more other nuclei are neither condensed nor show PH3 staining (Table 1). We have never seen asynchrony characterized by PH3 localization to some nuclei but not others in wildtype eggs or embryos at any stage through the end of the gonomeric division; wildtype meiotic products are always coordinated with respect to PH3 staining. PCNA localization in *Ya*^2 ^eggs and embryos also demonstrates asynchrony (Figure 6). In 24% of *Ya*^2 ^eggs and 17% of *Ya*^2 ^embryos some nuclei have PCNA localized to chromatin while other nuclei do not (Figure 6B, D); this asynchrony is not seen in wildtype eggs or embryos (Table 2).

**Table 1:** PH3 staining on chromatin of embryo nuclei.

**Chromatin phenotype**	***Ya***^2 ^**embryos**	**Wildtype embryos**
Apposed pronuclei synchronous	19	22
Apposed pronuclei asynchronous	8	0
Nuclear distribution other than apposed pronuclei: synchronous	109	197

Wildtype n = 219 embryos, *Ya*^2 ^n = 136 embryos.

**Table 2:** PCNA staining on chromatin of egg and embryo nuclei.

**Chromatin phenotype**	***Ya***^2 ^**embryos**	**Wildtype embryos**
Condensed, no PCNA	64%	60%
Decondensed, no PCNA	9%	13%
Decondensed, PCNA on chromatin	10%	27%
Asynchronous	17%	None

Wildtype n = 215 embryos, *Ya*^2 ^n = 168 embryos.

Our PCNA and PH3 immunofluorescence data highlight the nuclear asynchrony in YA-deficient embryos. Since cell-cycle asynchrony can occur in *Ya*^2 ^unfertilized eggs, the asynchrony in *Ya*^2 ^embryos is unlikely to be a distinction between maternally- and paternally-derived nuclei (although we cannot distinguish maternal from paternal chromatin in most *Ya*^2 ^embryos). In 0–15 minute collections of *Ya*^2 ^mutant embryos, individual nuclei can be at one of a few different cell cycle stages. These nuclei can be either condensed and mitotic-like as determined by the presence of phospho-histone H3 (Figure 5B), or they can be interphase-like decondensed nuclei (decondensed, but without PCNA, data not shown), or decondensed S-phase-like nuclei as indicated by the presence of PCNA (Figure 6C). We hypothesize that in the absence of YA function, the chromatin of egg and embryo nuclei can stain with PCNA and be somewhat decondensed before arriving at the ultracondensed chromatin phenotype.

### DNA replication occurs in YA-deficient eggs and embryos

**Figure 7:**
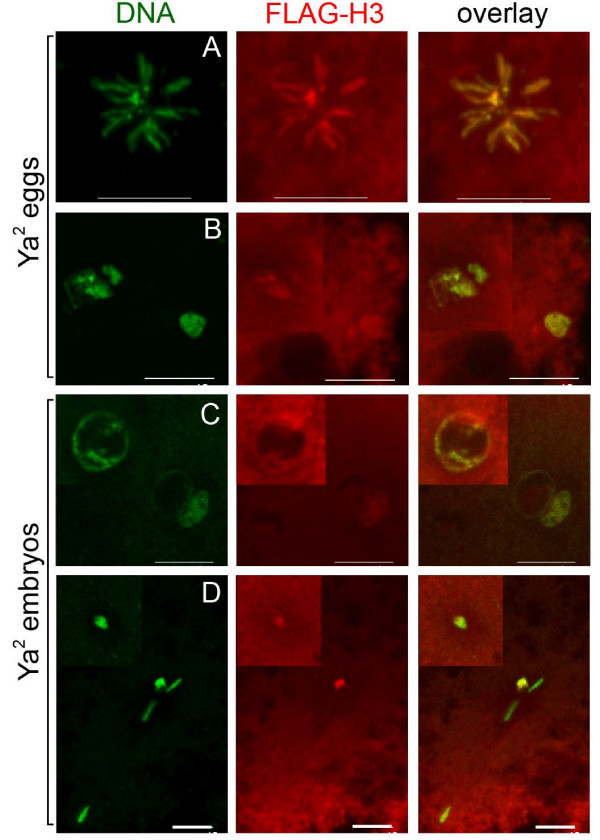
**Histone H3-FLAG distribution on chromatin of YA-deficient eggs and embryos**. Immunostaining of 0–15 minute old laid unfertilized H3-FLAG; *Ya*^2 ^eggs, and H3-FLAG;*Ya*^2 ^embryos. DNA shown in red, H3-FLAG in green. A-D are projections of multiple confocal images, with insets used to show nuclei in a greatly different focal plane. (A) YA-deficient egg with polar body rosette with H3-FLAG staining. (B) YA-deficient egg with four female meiotic products all with H3-FLAG staining. (C) YA-deficient embryo with two apposed nuclei and a third nearer the egg cortex, all with H3-FLAG staining. (D) YA-deficient embryo with five nuclei, two of which are more rounded and have H3-FLAG staining while three others do not. The H3-FLAG-staining nucleus not in the inset is closer to the three unstained nuclei along the Z axis than it is to the other H3-FLAG-staining nucleus shown in the inset. *Ya*^2 ^eggs n = 24, embryos n = 58. Bar = 10 μm.

The presence of PCNA on the chromatin of some YA-deficient eggs and embryos suggests they may undergo DNA replication. Because BrdU incorporation is undetectable in individual meiotic products ([26] and K.L.S. unpublished observations), to assay independently for replication we performed immunofluorescence using transgenic female flies bearing a FLAG-tagged version of canonical histone H3 [19], which is deposited on chromatin only in a replication-dependent process [53]. Maternally-derived histone H3-FLAG is first present on the sperm nucleus and is first visible on female meiotic products only after the first post-meiotic S phase in wildtype embryos [19]. Histone H3-FLAG is detectable on some nuclei (Figure 7) in the majority of YA-deficient eggs (54%) and embryos (93%), suggesting that replication has initiated in these embryos.

The incorporation of histone H3-FLAG into nuclei, like the presence of PCNA, indicates that DNA replication can occur in the absence of YA function. *Ya*^2 ^embryos are limited to at most one S phase prior to the gonomeric division, because the *Ya*^2 ^mutation prevents the multiple rounds of replication caused by *gnu *or *plu *mutations from occurring in double *Ya*^2^, *gnu *or *Ya*^2^, *plu *mutants [22,26]. Our results suggest that YA function may not be required for the pre-gonomeric S phase, but that cell cycle progression beyond the first mitosis, including additional rounds of S phase, requires embryos to proceed past the stage in which they are arrested in YA-deficient animals.

## Conclusion

Egg and sperm nuclei undergo significant developmental and cell cycle changes in the period between egg activation and the first mitosis of a fertilized egg. During this period, female meiosis must be completed and chromatin must be reorganized to transform the meiotic products into the female pronucleus and polar bodies, and the sperm nucleus into a male pronucleus [18]. Since the arrest point of *Ya*^2 ^mutant embryos is prior to the first mitosis, we examined here whether YA is needed for any of these events. Here we report that YA-deficient eggs and embryos appear to complete meiosis. Subsequently, the maternally-derived meiotic products and sperm nucleus lose their normal cell-cycle coordination and form abnormal associations, but these nuclei can still undergo at least some DNA replication. Because the arrest occurs after the end of meiosis and after the initiation of S phase, YA, or processes downstream of YA, appear to regulate molecules participating in the S-->(G2)--> M transition rather than those involved in the meiosis-->(G1)--->S phase transition.

The timing of the requirement for YA after egg activation is coincident with its localization to the nuclear lamina upon completion of meiosis, when the meiotic products decondense their chromatin [54,23]. Loss of nuclear identity, coupled with abnormal associations of haploid nuclei, is a phenotype that is consistent with disruption of nuclear envelope constituents. YA's ability to interact with chromatin would allow YA to sense, regulate, or transduce information concerning the chromatin condensation state. YA may function through its binding to lamin [23], DNA, and/or histone H2B [30] to detect and maintain a coordinated chromatin state of the male and female pronuclei appropriate for the gonomeric mitosis; in its absence cell cycle progression would be arrested due to the presence of asynchronous meiotic products or apposed pronuclei [55]. YA's interaction with chromatin may be appropriate for restoring chromatin synchrony to permit the gonomeric mitosis.

## Authors' contributions

JML did the FISH analysis, CLB did the in vitro activation studies, and KLS did the immunofluorescence analysis of cell cycle markers. MFW participated in and coordinated the design and interpretation of the study. KLS, JML and MFW wrote the manuscript with contributions from CLB. All authors read and approved the manuscript.

## Supplementary Material

Additional file 1 **Supplemental figures S1, S2, S3 and S4**. S1. Diagram of nuclear association patterns. S2. Chart of meiotic progression upon in vitro activation of wildtype eggs. S3. Western blots demonstrating normal egg activation in YA-deficient embryos. S4. FISH of embryo with fragmenting nuclei. Click here for file
