# Supplementary material for: YA is needed for proper nuclear organization to transition between meiosis and mitosis in Drosophila
Source: BMC Dev Biol. 2009 Jul 23;9:43. doi: 10.1186/1471-213X-9-43 (PMC2724486; doi:10.1186/1471-213X-9-43)
Supplement: Additional file 1 — Supplemental figures S1, S2, S3 and S4. S1. Diagram of nuclear association patterns. S2. Chart of meiotic progression upon in vitro activation of wildtype eggs. S3. Western blots demonstrating normal egg activation in YA-deficient embryos. S4. FISH of embryo with fragmenting nuclei. [file 1471-213X-9-43-S1.pdf]

<u>Supplemental Figure S1</u>:

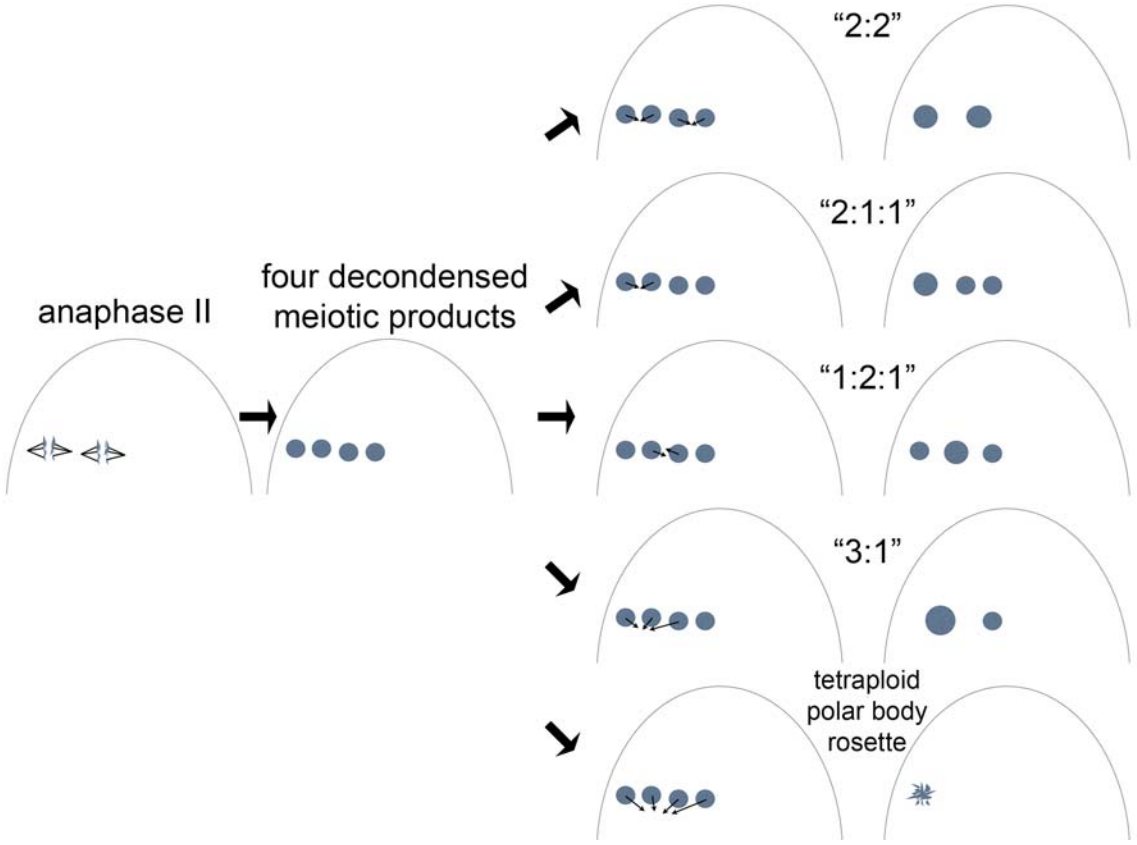

<u>Supplemental Figure S1</u>: Nuclear associations in unfertilized eggs.

The two meiosis II spindles are arranged in a line oriented perpendicular to the egg cortex [18,21]. After telophase II, the female meiotic products decondense their chromatin into a postmeiotic interphase-like state. The possible subsequent associations are illustrated on the right. Four haploid meiotic products, "3:1", and tetraploid polar body rosette are the only nuclear distributions seen in controls. These nuclear distributions are also present in Ya^2 eggs, along with the additional abnormal associations "2:2", "2:1:1" and "1:2:1".

<u>Supplemental Figure S2</u>:

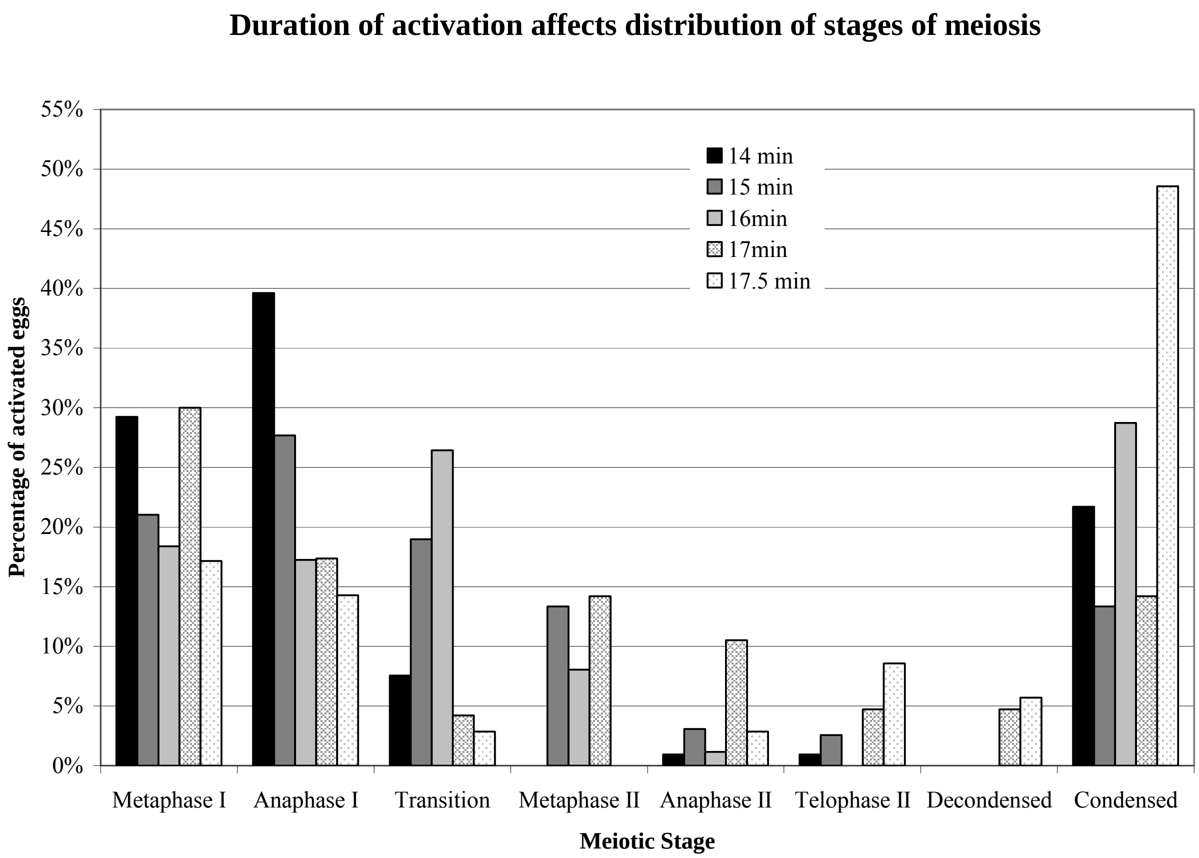

<u>Supplemental Figure S2</u>: Duration of activation of wildtype eggs affects distribution among meiotic stages.

Wildtype eggs were activated in vitro and staged based on DAPI staining. No error bars are shown because each time point is from a single experiment. Eggs with apparently condensed disorganized chromatin are grouped in the final category, although only some of the eggs in this category are likely to have completed successful meiosis.

Supplemental Figure S3:

A

Female genotype	Eggshell hardened	Number of eggs	Percent
control	131	131	100%
Ya^2	123	124	99%

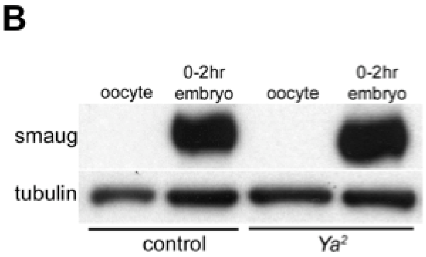

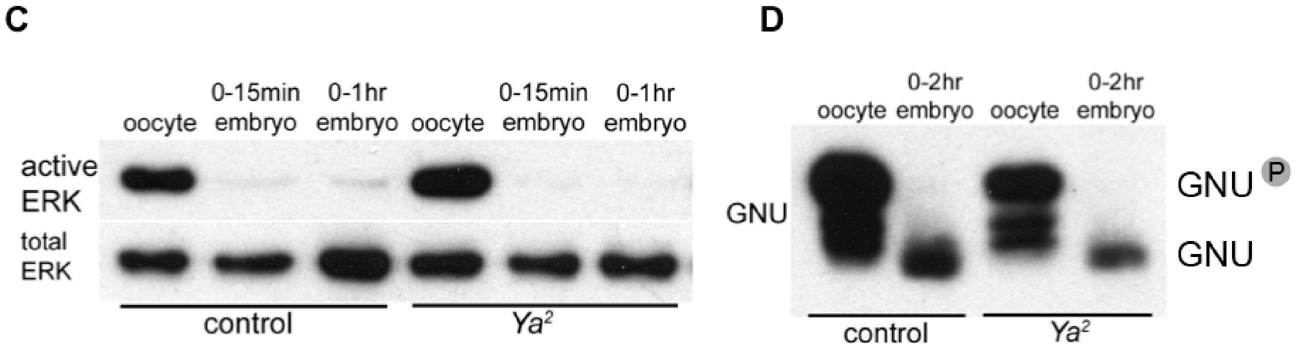

Supplemental Figure S3: **Four aspects of egg activation are normal in YA-deficient embryos.** (A) Laid embryos were immersed in 50% bleach for two minutes to test vitelline envelope hardening [1, 2]. The percentage of laid embryos impermeable to bleach was not significantly different between control and *Ya* mutant mothers. (B) Translation of the SMAUG (SMG) protein after egg activation was assayed by western blotting with anti-SMG antibody, a gift from W. Tadros and H. Lipshitz, diluted 1:10,000 [45]. The loading control, anti-α-tubulin antibody (Sigma, catalog #T5168), was diluted 1:10,000. SMG levels in 0-2hr-old embryos are not significantly different between control and *Ya* mutant. (C) Dephosphorylation of the MAP kinase ERK upon egg activation was assayed by western blotting with anti-phospho-ERK antibody [8]. Anti-total ERK was used as a loading control. Phospho-ERK levels decrease upon egg activation in *Ya* mutant embryos as in controls. (D) Dephosphorylation of GNU upon egg activation was assayed by western blotting with anti-GNU antibody, a gift from T. Orr-Weaver, diluted 1:5,000 [S1]. Phosphorylated GNU in oocytes has a slower electrophoretic mobility than dephosphorylated GNU in embryos [7], and the dephosphorylated form is present in *Ya* mutant embryos as in control embryos.

S1. Lee LA, Van Hoewyk D, Orr-Weaver TL: **The Drosophila cell cycle kinase PAN GU forms an active complex with PLUTONIUM and GNU to regulate embryonic divisions.** *Genes Dev* 2003, **17**:2979-91.

<u>Supplemental Figure S4</u>:

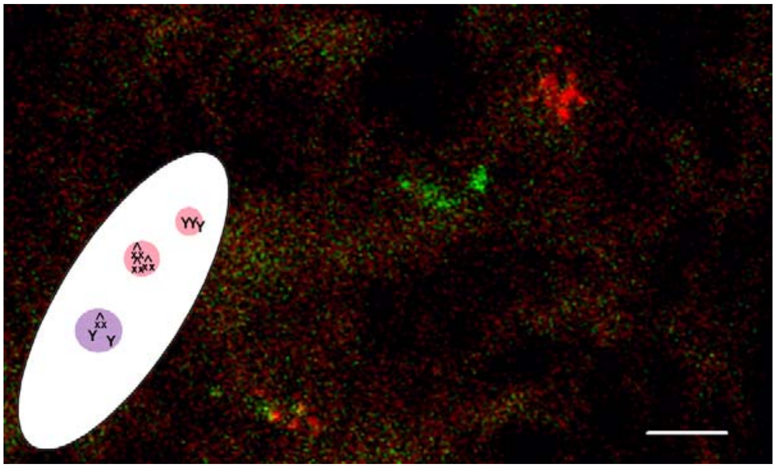

<u>Supplemental Figure S4</u>: FISH of embryo with fragmenting nuclei.

Three nuclei in which the ploidy is difficult to distinguish due to apparent deterioration of the nuclei as in [29]. The X chromosome probe's signal is green and the Y chromosome probe's is red. The inset illustration shows the orientation of the embryo and the positions of DAPI-stained nuclei inside. Bar =4um.
